# Hair Maintenance and Chemical Hair Product Usage as Barriers to Physical Activity in Childhood and Adulthood among African American Women

**DOI:** 10.3390/ijerph17249254

**Published:** 2020-12-10

**Authors:** Symielle A. Gaston, Tamarra James-Todd, Nyree M. Riley, Micaela N. Gladney, Quaker E. Harmon, Donna D. Baird, Chandra L. Jackson

**Affiliations:** 1 Epidemiology Branch, National Institute of Environmental Health Sciences, National Institutes of Health, Department of Health and Human Services, Research Triangle Park, NC 27709, USA; symielle.gaston@nih.gov (S.A.G.); nyree.riley@nih.gov (N.M.R.); micaelagladney@gmail.com (M.N.G.); quaker.harmon@nih.gov (Q.E.H.); baird@niehs.nih.gov (D.D.B.); 2 Department of Environmental Health, Harvard School of Public Health, Boston, MA 02115, USA; tjtodd@hsph.harvard.edu; 3 Department of Sociomedical Sciences, Mailman School of Public Health, Columbia University, New York, NY 10032, USA; 4 Intramural Program, National Institute on Minority Health and Health Disparities, Department of Health and Human Services, National Institutes of Health, Bethesda, MD 20814, USA

**Keywords:** hair preparations, African Americans, child, women, exercise

## Abstract

Qualitative studies have identified haircare practices as important culturally specific barriers to physical activity (PA) among Black/African American (AA) women, but quantitative investigations are lacking. Using the Study of Environment, Lifestyle and Fibroids data among 1558 Black/AA women, we investigated associations between hair product usage/hair maintenance behaviors and PA during childhood and adulthood. Participants reported childhood and current chemical relaxer and leave-in conditioner use. Self-reported PA included childhood recreational sports participation, leisure-time PA engagement during adulthood, and, at each life stage, minutes of and intensity of PA. Adjusting for socioeconomic and health characteristics, we used Poisson regression with robust variance to estimate prevalence ratios (PRs) and 95% confidence intervals (CIs) for each PA measure for more vs. less frequent hair product use/hair maintenance. Thirty-four percent reported ≥twice/year chemical relaxer use and 22% reported ≥once/week leave-in conditioner use at age 10 years, and neither were associated with PA at age 10 years. In adulthood, ≥twice/year chemical relaxer users (30%) were less likely (PR = 0.90 [95% CI: 0.79–1.02]) and ≥once/week leave-in conditioner users (24%) were more likely (PR = 1.09 [95% CI: 0.99–1.20]) to report intense PA compared to counterparts reporting rarely/never use. Hair product use/maintenance may influence PA among Black/AA women and impact cardiometabolic health disparities.

## 1. Introduction

Black or African-American (AA) adolescent girls and women have been consistently shown to have the highest prevalence of physical inactivity as well as obesity among United States (US) adolescent girls and women [1,2,3,4]. Regarding physical activity (PA), the Physical Activity Guidelines Advisory Committee recommends 60 min/day of PA for adolescents and ≥150 min/week of moderate PA or ≥75 min of vigorous PA for adults [5]. Recent data suggest that only 30% of Black/AA adolescent girls compared to 39% of White adolescent girls met PA guidelines five days per week, and only 37% of Black/AA women compared to 55% of White women engaged in the recommended amounts of PA per week [6]. Lack of physical activity is related to a variety of poor health outcomes, including mood disorders, obesity, and other markers of poor cardiometabolic health [7]. Given the association with poor cardiometabolic health outcomes for which Blacks/AAs are disproportionately affected [6], disparities in physical activity are of public health importance.

A potentially important, understudied contributor of racial/ethnic disparities in physical activity and relatedly, poor cardiometabolic health is the difference in hair product usage behaviors among Black/AA women compared to non-Black/AA US women [8,9,10]. Black/AA women generally have naturally curly hair that requires either permanent chemical straightening products or use of heat and temporary straightening products in order to obtain straight hair, which is a widely accepted European beauty standard [8,11,12]. These hair manipulation practices begin as early as childhood [9,13]. Hair straightening and other products marketed to and used mainly by Black/AA girls and women have been shown to contain chemicals with endocrine-disrupting properties [14]. The recent literature suggests that such chemicals are associated with poor cardiometabolic health outcomes through altering physiologic pathways like insulin secretion and adipogenesis [15]. Furthermore, likely resulting in synergistic effects on cardiometabolic health, hairstyles achieved by use of these products may be barriers to PA. To attain and maintain desired hairstyles, a significant investment of time and financial resources is made [13,16,17,18,19], and behaviors that counter these investments are avoided. For instance, moisture in the environment or from sweat can negatively impact such hairstyles. Indeed, “sweating out” hairstyles or causing hair to revert back to a naturally curly state during PA is often avoided [16,18,20,21,22,23]. Moreover, even if Black/AA women wear their hair in its naturally curly state, it is difficult for the hair to stay moisturized because the curl pattern makes it difficult for naturally produced oils to travel from the hair shaft down the strand [13,24]. Therefore, even wearing hair in a natural state can result in PA avoidance because natural hairstyles may still require extensive hair product use and hair maintenance. In fact, qualitative studies support hair maintenance as a unique barrier to physical activity among Black/AA women as early as adolescence and into adulthood [13,16,17,18,19,20,21,22,23,25,26,27].

Lack of physical activity throughout the life course may partially explain recalcitrant disparities in poor cardiometabolic health among AA/Black women, and further study of hair product usage and maintenance behaviors as contributors is warranted. Although there have been several qualitative or mixed-methods studies to date that have investigated the associations between hair maintenance and the lack of physical activity among Black/AA adolescent girls and women [13,16,17,18,19,20,21,22,23,25,26,27,28], quantitative studies with large sample sizes that consider chemical hair products usage patterns and physical activity are necessary. We sought to investigate the associations of (1) chemical hair product use and (2) hair maintenance behaviors with physical activity. We hypothesized inverse associations between chemical hair product use/hair maintenance frequency and physical activity. Given our previous findings in relation to changes in hair product use at different life stages among Black/AA women [9], we performed two cross-sectional investigations of these associations: one at age 10 years and one during adulthood.

## 2. Materials and Methods

### 2.1. Study of Environment, Lifestyle, and Fibroids

We used cross-sectional data from the Study of Environment, Lifestyle, and Fibroids (SELF), a prospective cohort study of 1693 women aged 23 to 35 years from the Detroit, Michigan area who self-identified as either Black/AA, alone or in combination with other racial/ethnic categories. The SELF was designed to investigate potential lifestyle and environmental risk factors for the development of uterine fibroids, and study details are described elsewhere [29]. Briefly, eligible SELF participants who were without a prior diagnosis of uterine fibroids were enrolled between January of 2010 and December of 2012. At enrollment, participants completed computer-assisted telephone and web interviews (CATI/CAWI) as well as clinic visits. Follow-up occurred at approximately 20-month intervals over 5 years. The current study used early life and adulthood data collected via CATI/CAWI. Early life data were collected at baseline (2010–2012), and adulthood data were collected at the second (2013–2014) or the third follow-up (2016–2018) if the participant missed the second follow-up. The institutional review board at the National Institute of Environmental Health Sciences and the Henry Ford Health System approved the SELF protocol, and each participant provided informed consent.

### 2.2. Study Participants

Among the cohort of 1693 participants, a total of 135 participants were sequentially excluded for either loss to follow-up before the adult hair product usage assessment (*n* = 130) or missing values for either childhood or adulthood relaxer or leave-in conditioner (*n* = 5). The final analytic sample comprised 1558 Black/AA women.

### 2.3. Hair Product Usage and Hair Maintenance Behaviors

#### 2.3.1. Childhood

At baseline, adult participants reported chemical hair product usage at age 10 years. Participants were asked, “How often was your hair treated with chemical products that change the texture of your hair, such as Jheri curl, relaxer, or perm when you were around 10 years old?” and reported use as *10 or more times a year, 5 to 9 times a year, 2 to 4 times a year, once a year*, or *rarely/never*. We combined responses to categorize chemical relaxer use as ≥twice/year, once/year, and rarely/never. Participants also reported leave-in conditioner use at age 10 years by responding to the question, “How often was your hair treated with leave-in conditioners or other hair products that remained on your hair rather than being rinsed out when you were around 10 years old?” Response options were *about every day, 3 to 5 times a week, 1 to 2 times a week, 1 to 3 times a month*, or *rarely or never*, and we categorized leave-in conditioner use as ≥once/week, 1–3 times/month, and rarely/never.

#### 2.3.2. Adulthood

During a follow-up interview, participants self-reported chemical relaxer use in the past 12 months by responding *12 or more times, 6–11 times, 2–5 times, once,* or *did not use in the past 12 months* to the following question, “During the past 12 months, about how often did you or someone else apply hair relaxers, straighteners, or perms to your hair?”. To standardize responses with the childhood categories, we categorized chemical relaxer use in the past 12 months as ≥twice/year, once/year, and rarely/never. Leave-in conditioner use in the past 12 months was assessed using two questions about type (i.e., rinse-out vs. leave-in) and frequency of conditioner use, and these questions are provided in a prior publication [9]. To standardize with childhood use, leave-in conditioner use in the past 12 months was categorized as ≥once/week, 1-3 times/month, and rarely/never.

Assessment of other hair products use in the previous 12 months is described in detail in a prior publication [9]. Briefly, participants also reported use of other hair products, and we used latent class analysis to identify latent classes based on frequency of use (i.e., high (≥once/week), medium (1–3 times/month), none (rarely/never)) of chemical relaxer/straightener, shampoo, individual growth/moisturizing products (i.e., shea butter, natural plant based oils, hair food, moisturizing creams and lotions, conditioners/detanglers), and a group of common hair styling products (e.g., hairspray or styling spritz, styling gel, mousse, pomade, hair grease, oil sheen, setting lotion). The model fit and interpretation for the three identified classes of hair product use were previously described [9]. Classes were labeled as following: Class One—High styling/low other product use; Class Two—High styling product/medium shampoo and conditioner use; and Class Three—High styling, shampoo, and growth/moisturizing product use (i.e., conditioner and oils).

### 2.4. Physical Activity (PA)

#### 2.4.1. Childhood

At baseline, participants self-reported PA at age 10 years. Participation in recreational sports at age 10 years was dichotomized as yes vs. no. Participants reported their average number of PA minutes on a typical weekday and a typical weekend day. We separately dichotomized weekday and weekend PA as ≥60 min/day (yes vs. no) based on the US Department of Health and Human Services guidelines for PA for children [5]. Among participants who engaged in any minutes of PA, which new PA guidelines suggest is beneficial for health [5], we assessed the intensity of PA. Participants reported how much of the time their PA was at a level high enough to cause a large increase in their breathing and heart rate. Response options included *very little or none of the time, less than half the time, about half the time,* and *more than half the time*. We created the following three categories for time spent engaging in intense PA: >half, ≤half, and very little/none of the time.

#### 2.4.2. Adulthood

At the second follow-up (2013–2014), participants reported PA in the past 12 months. Participants who missed the second follow-up were not asked about PA and were not included in the adulthood PA analysis (*n* = 118). Participation in leisure-time PA was based on either a yes or no response to three questions about playing sports, having a regular exercise routine or class, and participation in any other activities outside of exercise classes or regular workouts (e.g., biking, hiking, dancing, or other recreational activities). If a participant reported an affirmative response to any of the three questions, she was defined as participating in leisure-time PA. Participants then reported the typical amount of time per week they spent participating in the activities. We applied the US Department of Health and Human Services guidelines for PA among adults to dichotomize minutes of PA in adulthood as ≥150 min/week (yes vs. no) [5]. Lastly, participants who reported any minutes of PA also reported how much time during leisure-time PA that their activity level was high enough to cause a large increase in breathing and heart rate, and PA intensity categories were standardized with those of childhood (i.e., >half, ≤half, and very little/none).

### 2.5. Potential Confounders

Participants recalled childhood characteristics at age 10 years. Sociodemographic factors included the educational attainment of the mother or primary caregiver (≤high school or general education development (GED), some college or associate’s/technical degree, ≥bachelor’s degree), two-parent household (yes vs. no), household income during the majority of childhood (well-off, middle income, low income/poor), food insecurity at any time during childhood (yes vs. no), and neighborhood safety (very unsafe, somewhat unsafe, somewhat safe, very safe). Health behaviors and characteristics during childhood included weight status (heavier, same weight, lighter in comparison to other children) and enjoyment of physical activity (not at all/a little/somewhat, quite a bit/very much).

Adult sociodemographic factors that were assessed when PA was self-reported included age (years), educational attainment (≤high school/GED, some college or associate’s/technical degree, ≥bachelor’s degree), total annual household income (<$20,000, $20,000–$50,000, >$50,000), current full- or part-time employment (yes vs. no), and marital status (married/living with partner, divorced/separated/widowed, never married). Health behaviors and characteristics included body mass index (BMI) categories that were based on BMI calculated from objectively measured height and weight (underweight/normal weight [<25.0 kg/m^2^], overweight [25.0–29.9 kg/m^2^], class 1 obesity [30.0–34.9 kg/m^2^], class 2 and 3 obesity [≥35.0 kg/m^2^]), smoking status (never, former, current), alcohol consumption (none, moderate, heavy), good/very good/excellent general physical health (yes vs. no), and enjoyment of physical activity (not at all/a little bit/somewhat, quite a bit/very much).

### 2.6. Statistical Analysis

Study population characteristics during childhood and adulthood were described as counts and percentages or as means and standard deviations. Using Poisson regression models with robust variance [30], we estimated prevalence ratios (PRs) and 95% confidence intervals (CIs) for each PA outcome during childhood or adulthood, separately, among (1) participants who reported more frequent chemical relaxer or leave-in conditioner use, separately, compared to those who reported rarely/never using each and (2) participants who reported high styling, shampoo, and growth/moisturizing product use (Class Three) and high styling product/medium shampoo and conditioner use (Class Two) compared to participants with high styling/low other product use (Class One). We determined potential confounders to include in the adjusted models based on the previous literature and our construction of directed acyclic graphs [19,22,31,32,33,34,35,36].

All statistical models were estimated sequentially. For the analysis at age 10 years, Model 1 was unadjusted, Model 2 was adjusted for socioeconomic characteristics (childhood household educational attainment, two-parent household, childhood household income, and childhood food insecurity), Model 3 was additionally adjusted for neighborhood safety, and Model 4 was additionally adjusted for weight status relative to peers. For the adulthood analysis among participants with data for PA outcomes in the past 12 months (*n* = 1440), Model 1 was unadjusted, Model 2 was adjusted for sociodemographic characteristics (age, educational attainment, annual household income, employment status, and marital status), Model 3 was additionally adjusted for health behaviors/characteristics (smoking status, alcohol use, BMI), and Model 4 was additionally adjusted for enjoyment of PA, which may affect hair styling and maintenance as well as PA engagement, but could also act as a mediator on the pathway to PA. SAS, version 9.4 for Windows (Cary, NC, USA) was used in the analyses.

### 2.7. Sensitivity and Potential Modification Analyses

To test model assumptions about including enjoyment of PA as a potential confounder or mediator versus an effect modifier, we stratified fully adjusted adulthood models by enjoyment of PA (yes [quite a bit/very much] vs. no [not at all/a little bit/somewhat]). Secondly, since barriers to physical activity may differ by hairstyle or whether participants wear their hair naturally or in a relaxed/straightened state [25], we stratified fully adjusted models for leave-in conditioner use and hair maintenance behaviors in adulthood by whether participants reported chemical relaxer use in the past 12 months (yes [any use] vs. no [rarely/never]). In additional analyses, we stratified fully adjusted models for adulthood chemical hair product use/hair maintenance behaviors and each PA outcome in adulthood by the following potential modifiers: age group dichotomized at the median (<33 years vs. ≥33 years), dichotomized annual household income (≤$50,000 vs. >$50,000), and dichotomized obesity status (non-obese vs. obese). Age category was considered a potential modifier because studies suggest attitudes about hairstyles and maintenance practices may vary by generation and/or age as the social environment has changed over time [9,12,37]. Studies also suggest hair product use/maintenance practices and PA engagement may vary by income [3,4,9]. Lastly, BMI category may act as an effect modifier if propensity towards “sweating out” hairstyles vary by obesity status along with PA engagement [3,4].

## 3. Results

### 3.1. Study Population

During early life, approximately half of participants (47%) resided in a household where the highest educational attainment was ≤high school, lived in middle-income households (53%), and lived in two-parent households (52%) (Table 1). One-third (34%) reported chemical relaxer use at least twice/year while 22% reported leave-in conditioner use at least once/week. Approximately half (49%) reported participation in sports, and most reported ≥60 min of PA on weekdays (89%) and weekends (92%). Among 1547 participants who reported any minutes of PA, 30% reported spending more than half of the time engaging in intense PA.

At the time of data collection, the mean age and standard deviation of participants was 33 ± 3.4 years (Table 2). Most participants (84%) attained at least some college/associate/or technical degree, 25% had an annual household income of >$50,000, and 78% were employed either full- or part-time. Furthermore, 65% of participants were obese, 85% were in good/very good/excellent general physical health, and 44% reported quite a bit or very much enjoyment of PA. Approximately 30% of participants reported chemical relaxer use at least twice/year and 24% reported leave-in conditioner at least once/week in the past 12 months. Frequency of chemical relaxer and leave-in conditioner use were not associated, and only 7% of participants reported the most frequent usage category of both (Supplementary Table S1). Regarding hair maintenance in the past 12 months, 36% had high styling/low other product use; 33% high styling product/medium shampoo and conditioner use; and 31% high styling, shampoo, and growth/moisturizing product use. Over half of women (59%) participated in leisure-time PA, 30% met PA guidelines, and among those who reported any minutes of PA (*n* = 817), 42% reported spending more than half of the time engaged in intense PA in the past 12 months. Adulthood characteristics of participants by chemical relaxer use, leave-in conditioner use, and hair maintenance behaviors/hair product use in the prior 12 months are described in Supplementary Tables S2–S4.

### 3.2. Chemical Hair Product Use and PA in Childhood

Neither chemical relaxer use nor leave-in conditioner use at age 10 years was associated with participation in recreational sports, ≥60 min/day of PA on weekdays or weekends, or PA intensity at age 10 years (Table 3).

### 3.3. Chemical Hair Product Use, Hair Maintenance Behaviors, and PA in Adulthood

Prior to adjustment, both chemical relaxer and leave-in conditioner use were associated with PA in the previous 12 months (Table 4). However, associations attenuated after adjustment. Nonetheless, after adjustment for sociodemographic characteristics, health behaviors and characteristics, and enjoyment of PA, participants who reported using relaxers the most frequently (≥twice/year) were 10% less likely to participate in leisure-time PA (PR = 0.90 [95% CI: 0.82–1.00]) and, among those with any minutes of PA, were marginally less likely to report spending more than half the time engaged in PA (PR = 0.90 [0.79–1.02]) compared to participants who reported rarely/never using chemical relaxer. Conversely, more frequent vs. rarely/never using leave-in conditioner was positively associated with participation in leisure-time PA, meeting PA guidelines (≥150 min/week of leisure-time PA), and PA intensity prior to adjustment. After full adjustment, associations were attenuated but remained suggestive of the higher prevalence of PA outcomes among more frequent users of leave-in conditioner. For instance, compared to participants who reported rarely/never using leave-in conditioner, participants who reported leave-in conditioner use 1–3 times per month had an 18% higher prevalence of meeting PA guidelines (PR = 1.18 [0.98–1.43]) and, among participants with any minutes of PA, participants who reported ≥once/week use had a 9% higher prevalence of reporting spending more than half of the time engaging in intense PA (PR = 1.09 [0.99–1.20]) after full adjustment.

Compared to participants with high styling/low other product use, participants with high styling product/medium shampoo and conditioner use and participants with high styling, shampoo, and growth/moisturizing product use were more likely to report participation in leisure-time PA, meeting PA guidelines, and intense PA > half the time (among participants with any minutes of PA) in unadjusted models (Table 5). After full adjustment, participants who reported high styling, shampoo, and growth/moisturizing product use (Class Three) versus high styling/low other product use (Class One) had a 23% higher prevalence of engaging in ≥150 min/week of PA (PR = 1.23 [95% CI: 1.02–1.49]).

### 3.4. Sensitivity and Potential Modification Analyses

The sensitivity analysis did not support enjoyment of PA as a potential effect modifier (Supplementary Table S5). After stratification by chemical relaxer use in the previous 12 months, some strata had small sample sizes, leading to a reduction in precision, and although confidence intervals overlapped, estimates differed by chemical relaxer use for certain PA outcomes (Supplementary Table S6). For instance, participants without chemical relaxer use who used leave-in conditioner ≥once/week had 17% higher prevalence of intense PA >half the time (PR = 1.17 [1.06–1.30]) compared to their counterparts who rarely/never used leave-in conditioner, but there was no association among participants who reported chemical relaxer use in the previous 12 months (PR = 0.89 [95% CI:0.69–1.15]). Furthermore, participants without relaxer use who reported high styling, shampoo, and growth/moisturizing product use and high styling product/medium shampoo and conditioner use were more likely to report meeting PA guidelines compared to participants who reported high styling/low other product use (PR = 1.43 [1.11–1.85] and PR = 1.34 [1.04–1.73]). However, participants with relaxer use who reported high styling, shampoo, and growth/moisturizing product use and participants with high styling product/medium shampoo and conditioner use were no more likely to report meeting PA guidelines than participants who reported high styling/low other product use (PR = 1.03 [0.76–1.38] and PR = 0.94 [0.69–1.27]).

After stratification by age group, annual household income, and obesity status, sample size in some strata reduced both precision and our ability to detect potential effect modification; however, age group and obesity status may act as effect modifiers (Supplementary Tables S7–S9). For instance, medium use (1–3 times/month) versus rarely/never use of leave-in conditioner was associated with a higher prevalence of participation in leisure-time PA among participants aged <33 years, but was not associated among participants aged ≥33 years (p-interaction<0.10). Additionally, although not observed among participants aged <33 years, ≥twice/year versus rarely/never using chemical relaxer was associated with a lower prevalence of meeting PA guidelines among participants aged ≥33 years (p-interaction<0.05). Lastly, although there was no association among obese participants, non-obese participants who reported high styling, shampoo, and growth/moisturizing product use were suggestively more likely to report spending over half the time engaged in intense PA compared to their counterparts who reported high styling/low other product use (p-interaction<0.10).

## 4. Discussion

In this large sample of Black/AA women, we evaluated associations between chemical hair product usage as well as hair maintenance and PA in childhood and adulthood. Hair product use was not associated with childhood PA. Instead, we found chemical relaxer use was a potential barrier to PA in adulthood, with greater chemical relaxer use in adulthood being associated with a lower prevalence of leisure-time PA, meeting PA guidelines, and spending at least half the time engaged in intense PA prior to adjustment. The associations with leisure-time PA, though attenuated, and a marginal association with intense PA held even after adjustment for key sociodemographic factors and health behaviors. Furthermore, Black/AA women who frequently vs. rarely used leave-in conditioner and who engaged in more versus fewer hair maintenance behaviors were more likely to meet PA guidelines in adulthood. The associations between greater hair maintenance and higher levels of PA were suggestively stronger among Black/AA women who did not use chemical relaxers in the previous 12 months.

Overall, our results regarding chemical relaxer use in adulthood are consistent with the previous literature. Consistent with the findings from prior qualitative studies that suggested hair maintenance may act as a barrier to PA among Black/AA women in adulthood [16,17,18,19,21,22,25], we found that women who reported frequent versus rare/no chemical relaxer use in adulthood were less likely to engage in both leisure-time and intense PA. However, unlike prior studies [13,23,26,27], our results did not support chemical relaxer use as a barrier to childhood PA. This inconsistent finding is likely related to the different age of assessment across studies. Although our analysis corresponded to late childhood/pre-adolescence (age 10 years), prior studies were among Black/AA adolescents [13,23,26,27], which is a life stage when girls become more concerned about physical appearance and likely engage in different hair product use/hair maintenance behaviors. Additional quantitative studies among Black/AA children and adolescents are needed.

Our results for associations with leave-in conditioner use and hair maintenance may be related to cultural shifts related to hair and PA. We found that women who reported more frequent leave-in conditioner use and hair maintenance were more likely to meet PA guidelines compared to women who reported rarely/no leave-in conditioner use and less hair maintenance. While these results appear to be contrary to the previous literature suggesting hair maintenance as a barrier to PA [16,17,18,19,21,22,25,28], it is likely that our finding is related to the cross-sectional nature of our study and the natural hair movement or recent shift towards wearing hair in its naturally curly state as well as the increasing community support (e.g., online hair communities) and ability to care for natural hair while maintaining physical activity among Black/AA women that may not be reflected in some of the previous literature [12,17,18,28,38]. Hair maintenance practices captured in SELF occurred years after the onset of the natural hair movement, while previous studies that provided dates of data collection captured practices largely before or during the beginning stages of the movement. Although confidence intervals overlapped in our sensitivity analysis, hair maintenance was not associated with PA among women who reported chemical relaxer use, but hair maintenance was positively associated with PA among women who reported no chemical relaxer use. Among women with natural hair, those with greater hair maintenance may be more likely to meet PA guidelines and engage in intense PA compared to women with lower hair maintenance. This observation may result from potential reverse causation where women who engage in more PA spend more time doing the hair maintenance activities required, including washing hair, using leave-in conditioner, and styling natural hair, as a part of appearance management following PA. Black/AA women with natural hair may not as strongly view hair maintenance as a barrier to PA because natural hair maintenance may be less costly and time-intensive. This possibility is dependent upon hair style, approaches women use to maintain their hair, and whether they style their own hair or have it professionally styled. For instance, a prior study among Black/AA women cosmetologists/hair stylists or their clients (aged 18–71 years) who all wore natural hair reported a low prevalence of PA [28]. Their results could be related to the age range of participants as well as to the possibility that hair maintenance can remain a barrier even among women who wear their hair naturally because of the financial and time investments related to professional styling and preferred hair styles (e.g., temporarily straightened hair) that are hard to maintain with PA [17]. Additional studies that assess non-professional (e.g., at home) versus professional styling are warranted to investigate natural hair styles/styling practices in relation to PA and other potential hypotheses across additional populations of Black/AA women.

Several characteristics of the social environment may explain our results. Traditionally, Black/AA women have used chemical straighteners to achieve straight hairstyles that were deemed socially desirable and acceptable [8,12,20]. In order to maintain socially desirable straight hairstyles, behaviors like PA were avoided in order to not “sweat out” the hair or ruin desired hairstyles that required considerable time and financial investments [16,18,20,21,22,23]. Therefore, the social environmental influence of traditional ideal beauty standards related to hair acted as an upstream determinant or one of the fundamental causes of the observed association between frequent chemical relaxer use and lack of physical inactivity. In recent years, wearing of natural hair among Black/AA women has become more culturally acceptable, and there have been changes in the social environment such as the passing of hair anti-discrimination policies including the Creating a Respectful and Open World for Natural Hair (CROWN) Act that prohibits discrimination based on hairstyles and hair texture in schools and workplaces, which has been passed in seven US states and by the US House of Representatives, to date [12,39]. Embracing natural hair is reflective in our current sample. We previously found that at age 15 years, 17% of participants did not use chemical relaxers/straighteners, and non-usage of chemical relaxers/straighteners increased to 59% during adulthood [9]. Often, wearing natural hair allows Black/AA women to style and maintain their own hair and to not have to frequently use professional hair stylists. With the ability to maintain their own hair, the financial implications and costs related to maintaining their hair may be reduced, thus potentially reducing hair maintenance as a barrier to PA. Therefore, the recent natural hair movement and embracing of natural hair among women in the Black/AA community may have resulted in a reduction of hair maintenance as a barrier to PA.

Also of importance, hair straightening and other products marketed to and used by Black/AA girls and women have been shown to contain chemicals with endocrine-disrupting properties that have been implicated as contributors to poor cardiometabolic health outcomes [14,15]. The combination of exposure to endocrine-disrupting chemicals and the alteration of physical activity related to chemical hair product usage/hair maintenance behaviors may synergistically contribute to disparities in poor cardiometabolic health among Black/AA women over the life course, making this group of US women particularly vulnerable. This population would benefit from future research that considers hair product formulations, hair product use, and health behaviors related to hair product use as important, overarching determinants of cardiometabolic health that can serve as targets for intervention. Furthermore, in our additional analyses, we observed that relationships between hair product use/maintenance may vary by age and obesity status. Each of these characteristics require additional investigation as they may aid in the further identification of particularly vulnerable populations within the community of Black/AA women.

Our study should be interpreted in the context of its limitations and strengths. The cross-sectional study design as well as the assessment of hair product use and PA at one time point in adulthood leads to the possibility of reverse causality. For example, greater PA may have resulted in greater hair maintenance. Other limitations include our use of subjective versus objective measurements of hair product use and PA, which may lead to non-differential misclassification. Furthermore, there is potential for recall bias of childhood behaviors at age 10 years among adults. Longitudinal studies over the life course are warranted. Additionally, we assessed leisure-time PA during adulthood and may have underestimated total PA. Nonetheless, by focusing on leisure-time PA, our results better elucidate hair maintenance as a barrier to PA that is engaged in by choice rather than as a necessity in the context of work. Our results may also be non-generalizable to other populations because our study participants were limited to one geographic location, and age was relatively homogenous within the cohort of reproductive age Black/AA women. Results may vary across different populations of Black/AA women in different US regions since beauty practices may vary by region. Results also may vary by birthplace/immigration status and generation because culture and beauty standards change over time. This study should be replicated in other groups. Nonetheless, the strengths of our study include its large sample size of an understudied population, our detailed assessment of hair product usage during both childhood and adulthood, and the examination of several measures of PA.

## 5. Conclusions

The common observation of chemical relaxer use as a barrier to PA among Black/AA women in qualitative studies was supported by this novel quantitative study. Wearing natural hair may reduce hair maintenance as a barrier to PA among Black/AA women. With Black/AA women having a higher prevalence of obesity compared to non-Hispanic White women, identifying modifiable factors that contribute to these disparities is imperative. Further investigation of culturally relevant yet understudied barriers to PA, including hair product use and hair maintenance, in the Black/AA community can inform intervention targets that can contribute to the elimination of cardiometabolic health disparities among this vulnerable group of women.

## Figures and Tables

**Table 1:** Early life characteristics of participants at age 10 years, Study of Environment, Lifestyle and Fibroids (SELF) (2010–2012 and 2014, *N* = 1558).

	*n*	%
**Sociodemographic Characteristics**		
Highest Educational Attainment of Primary Caregiver		
≤High School	727	47
Some College/Associate or Technical Degree	650	42
≥Bachelor’s Degree	179	11
Household Income during Most of Childhood		
Well off	112	7
Middle Income	822	53
Low Income/Poor	623	40
Two-parent Household at Age 10 Years (Yes)	809	52
Food Insecurity during Childhood (Yes)	193	12
Neighborhood Safety		
Very Unsafe	79	5
Somewhat Unsafe	262	17
Somewhat Safe	696	45
Very Safe	521	>33
**Health Behaviors and Characteristics**		
Weight Status in Comparison to Other Children		
Heavier	431	28
Same Weight	587	38
Lighter	540	35
Enjoyment of Physical Activity		
Not at All/A Little/Somewhat	255	16
Quite a Bit/Very Much	1303	>84
**Hair Product Usage/Hair Maintenance**		
Chemical Relaxer/Straightener at age 10 Years		
≥Twice/Year	536	34
Once/Year	170	11
Rarely/Never	852	55
Leave-in Conditioner at age 10 Years		
≥Once/Week	348	22
1–3 Times/Month	537	34
Rarely/Never	673	43
**Physical Activity (PA)**		
Participate in Sports (Yes)	770	49
≥60 Minutes/Day of PA on School Days/Weekdays (Yes)	1389	89
≥60 Minutes/Day of PA on Weekends/Vacation (Yes)	1426	92
Percentage of Time Spent Engaging in Intense PA *		
>Half	461	30
≤Half	745	48
Very Little/None	341	22

Note: Percentages may not sum to 100 due to missing values and rounding; Missing values: highest educational attainment in household = 2; household income = 1; two-parent household = 15; food insecurity = 1; played sports = 3; weekday PA = 8; weekend PA = 3; * Percentage of time spent engaging in intense PA was assessed among the 1547 participants who engaged in any minutes of PA weekdays or weekends, on average.

**Table 2:** Adulthood characteristics of participants, Study of Environment, Lifestyle and Fibroids (SELF 2013–2014, *N* = 1440) *.

	*n*	%
**Sociodemographic Characteristics**		
Age, years (mean ± SD)	33 ± 3.4	
Educational Attainment		
≤High School	223	15
Some College or Associate’s/Technical Degree	740	51
≥Bachelor’s Degree	476	33
Total Annual Household Income		
<$20,000	511	35
$20,000–$50,000	563	39
>$50,000	364	25
Current Full- or Part-Time Employment (yes)	1120	78
Marital Status		
Married/Living with Partner	588	41
Divorced/Separated/Widowed	279	19
Never Married	573	40
**Health Behaviors and Characteristics**		
BMI (kg/m^2^)		
Underweight/Normal (<25.0)	225	16
Overweight (25.0 to 29.9)	279	19
Obese Class I (30.0–34.9)	286	20
Obese Classes II and III (≥35.0)	650	45
Smoking Status		
Never	1003	70
Former	134	9
Current	303	21
Alcohol Use (Past 12 Months)		
None	206	14
Moderate	961	67
Heavy	273	19
Good/Very Good/Excellent General Physical Health (Yes)	1217	85
Enjoyment of Physical Activity		
Not at All/A Little/Somewhat	812	56
Quite a Bit/Very Much	628	44
**Hair Product Use/Hair Maintenance**		
Chemical Relaxer/Straightener		
≥Twice/Year	426	30
Once/Year	171	12
Rarely/Never	843	58
Leave-in Conditioner		
≥Once/Week	345	24
1–3 Times/Month	284	20
Rarely/Never	811	56
Hair Maintenance Behaviors		
Class 1: High Styling Product Use/Low Use Other Products	512	36
Class 2: High Styling Product Use/Medium Shampoo and Conditioners	485	33
Class 3: High Styling Product, Growth/Moisturizing Product, and Shampoo Use	443	31
**Physical Activity (PA) in the Past 12 Months**		
Participation in regular leisure time PA (sports, took exercise class/did other activities [e.g., biking, hiking, dancing]) (yes)	848	59
≥150 Minutes/Week of Regular Leisure-time Physical Activity (not including walking) (yes)	438	30
Percentage of Time Spent Engaging in Intense Physical Activity **		
>Half	347	42
≤Half	364	45
Very Little/None	106	13

Abbreviations: SD (Standard Deviation); BMI (body mass index); kg (kilograms); m (meters). Note: Percentages may not sum to 100 due to missing values; Missing Values: educational attainment = 1; household income = 2; general health = 9; * 118 of the 1558 participants were lost to follow-up and did not provide adulthood PA data; ** Percentage of time spent engaging in intense PA was assessed among the 817 participants who engaged in any minutes of PA per week, on average.

**Table 3:** Prevalence ratios for engagement in physical activity (PA) at age 10 for participants who reported chemical relaxer or leave-in conditioner use compared to participants who reported rarely/never use, Study of Environment, Lifestyle and Fibroids (*N* = 1558).

	Prevalence Ratio (95% CI)			
**Chemical Hair Product Usage**	**Model 1**	**Model 2**	**Model 3**	**Model 4**
**Chemical Relaxer**				
	**Participates in Recreational Sports (yes vs. no)**			
≥Twice/Year vs. Rarely/Never	0.91 (0.81–1.01)	0.91 (0.82–1.02)	*0.91 (0.81–1.02)*	0.92 (0.83–1.03)
Once/Year vs. Rarely/Never	0.89 (0.74–1.06)	0.90 (0.75–1.08)	0.90 (0.75–1.07)	0.91 (0.76–1.08)
	**≥60 Minutes/Day of Weekday PA (yes vs. no)**			
≥Twice/Year vs. Rarely/Never	1.00 (0.97–1.04)	1.00 (0.97–1.04)	1.00 (0.97–1.04)	1.00 (0.97–1.04)
Once/Year vs. Rarely/Never	1.00 (0.94–1.06)	1.00 (0.95–1.06)	1.00 (0.95–1.06)	1.00 (0.95–1.06)
	**≥60 Minutes/Day of Weekend PA (yes vs. no)**			
≥Twice/Year vs. Rarely/Never	1.01 (0.98–1.05)	1.01 (0.98–1.05)	1.01 (0.98–1.05)	1.02 (0.99–1.05)
Once/Year vs. Rarely/Never	0.98 (0.92–1.03)	0.98 (0.93–1.03)	0.98 (0.93–1.03)	0.98 (0.93–1.04)
	Percentage of Time Spent Engaging in Intense PA (≤Half vs. Very Little/None) *			
≥Twice/Year vs. Rarely/Never	1.04 (0.96–1.13)	1.05 (0.97–1.15)	1.05 (0.96–1.14)	1.03 (0.95–1.12)
Once/Year vs. Rarely/Never	0.93 (0.81–1.08)	0.96 (0.83–1.11)	0.95 (0.82–1.10)	0.94 (0.81–1.09)
	Percentage of Time Spent Engaging in Intense PA (>Half vs. Very Little/None) *			
≥Twice/Year vs. Rarely/Never	1.07 (0.94–1.21)	1.07 (0.95–1.21)	1.06 (0.94–1.20)	1.07 (0.94–1.21)
Once/Year vs. Rarely/Never	0.92 (0.74–1.14)	0.95 (0.77–1.16)	0.94 (0.76–1.15)	0.94 (0.77–1.16)
**Leave-in Conditioner**				
	**Participates in Recreational Sports (yes vs. no)**			
≥Once/Week vs. Rarely/Never	1.11 (0.98–1.27)	1.10 (0.96–1.25)	1.09 (0.96–1.24)	1.09 (0.96–1.24)
1–3 Times/Month vs. Rarely/Never	1.06 (0.94–1.19)	1.06 (0.94–1.19)	1.06 (0.94–1.19)	1.06 (0.95–1.19)
	**≥60 Minutes/Day of Weekday PA (yes vs. no)**			
≥Once/Week vs. Rarely/Never	0.99 (0.95–1.04)	1.00 (0.95–1.04)	1.00 (0.95–1.04)	1.00 (0.96–1.05)
1–3 Times/Month vs. Rarely/Never	1.01 (0.97–1.05)	1.01 (0.97–1.05)	1.01 (0.97–1.05)	1.01 (0.97–1.05)
	**≥60 Minutes/Day of Weekend PA (yes vs. no)**			
≥Once/Week vs. Rarely/Never	1.00 (0.96–1.04)	1.01 (0.97–1.05)	1.01 (0.97–1.05)	1.01 (0.97–1.05)
1–3 Times/Month vs. Rarely/Never	0.99 (0.95–1.02)	0.99 (0.95–1.02)	0.99 (0.95–1.02)	0.99 (0.96–1.02)
	Percentage of Time Spent Engaging in Intense PA (≤Half vs. Very Little/None) *			
≥Once/Week vs. Rarely/Never	0.94 (0.84–1.04)	0.95 (0.85–1.06)	0.95 (0.85–1.06)	0.96 (0.86–1.06)
1–3 Times/Month vs. Rarely/Never	1.00 (0.92–1.10)	1.02 (0.93–1.11)	1.02 (0.93–1.11)	1.01 (0.93–1.11)
	Percentage of Time Spent Engaging in Intense PA (>Half vs. Very Little/None) *			
≥Once/Week vs. Rarely/Never	0.91 (0.77–1.06)	0.94 (0.80–1.10)	0.92 (0.79–1.08)	0.94 (0.80–1.10)
1–3 Times/Month vs. Rarely/Never	0.98 (0.86–1.13)	1.02 (0.89–1.16)	1.01 (0.88–1.15)	1.01 (0.89–1.16)

Model 1: unadjusted; Model 2: adjusted for socioeconomic factors (childhood household educational attainment, two-parent household, childhood household income, and childhood household food insecurity); Model 3: Model 2 + neighborhood safety; and Model 4 = Model 3 + weight status. Italicized values indicate statistical significance at a two-sided *p*-value < 0.10. Abbreviations: CI (confidence interval). Missing values: highest educational attainment in household = 2; two-parent household = 15; household income = 1; food insecurity = 1; played sports = 3; weekday PA = 8; weekend PA = 3. * Percentage of time spent engaging in intense PA was assessed among the 1547 participants who engaged in any minutes of PA weekdays or weekends, on average.

**Table 4:** Prevalence ratios for engagement in physical activity (PA) for participants who reported chemical relaxer or leave-in conditioner use in the past 12 months compared to participants who reported rarely/never use, Study of Environment, Lifestyle and Fibroids (*N* = 1440 *).

	Prevalence Ratio (95% CI)			
Chemical Hair Product Use	Model 1	Model 2	Model 3	Model 4
**Chemical Relaxer**	**Participation in Leisure-time PA (yes vs. no)**			
≥Twice/Year vs. Rarely/Never	**0.84 (0.76–0.94)**	**0.88 (0.80–0.98)**	**0.89 (0.80–0.99)**	**0.90 (0.82–1.00)**
Once/Year vs. Rarely/Never	0.92 (0.80–1.06)	1.01 (0.88–1.17)	1.02 (0.89–1.18)	1.00 (0.87–1.14)
	**≥150 Minutes/Week of Leisure-time PA (yes vs. no)**			
≥Twice/Year vs. Rarely/Never	**0.81 (0.68–0.98)**	0.88 (0.73–1.06)	0.88 (0.73–1.06)	0.90 (0.75–1.08)
Once/Year vs. Rarely/Never	0.98 (0.77–1.25)	1.13 (0.89–1.44)	1.14 (0.89–1.45)	1.09 (0.87–1.38)
	**Percentage of Time Spent Engaging in Intense PA** (≤Half vs. Very Little/None) **			
≥Twice/Year vs. Rarely/Never	1.00 (0.89–1.11)	1.01 (0.91–1.12)	0.99 (0.89–1.10)	0.99 (0.89–1.10)
Once/Year vs. Rarely/Never	1.03 (0.90–1.19)	1.11 (0.97–1.27)	1.11 (0.97–1.28)	1.10 (0.96–1.27)
	**Percentage of Time Spent Engaging in Intense PA** (>Half vs. Very Little/None) **			
≥Twice/Year vs. Rarely/Never	**0.85 (0.73–0.98)**	0.90 (0.79–1.02)	*0.90 (0.79–1.02)*	*0.90 (0.79–1.02)*
Once/Year vs. Rarely/Never	0.88 (0.72-1.07)	1.06 (0.90–1.26)	1.04 (0.87–1.23)	1.02 (0.86–1.21)
**Leave-in Conditioner**	**Participation in Leisure-time PA (yes vs. no)**			
≥Once/Week vs. Rarely/Never	**1.14 (1.03–1.26)**	*1.09 (0.99–1.20)*	1.08 (0.98–1.19)	1.07 (0.98–1.18)
1–3 Times/Month vs. Rarely/Never	**1.15 (1.03–1.28)**	1.09 (0.98–1.21)	1.07 (0.97–1.19)	1.09 (0.98–1.21)
	**≥150 Minutes/Week of Leisure-time PA (yes vs. no)**			
≥Once/Week vs. Rarely/Never	1.25 (1.04–1.50)	1.17 (0.97–1.40)	1.13 (0.94–1.35)	1.13 (0.94–1.34)
1–3 Times/Month vs. Rarely/Never	1.26 (1.04–1.53)	1.17 (0.96–1.42)	1.15 (0.95–1.39)	*1.18 (0.98–1.43)*
	**Percentage of Time Spent Engaging in Intense PA** (≤Half vs. Very Little/None) **			
≥Once/Week vs. Rarely/Never	**1.15 (1.03–1.28)**	1.07 (0.96–1.18)	1.04 (0.94–1.16)	1.04 (0.94–1.15)
1–3 Times/Month vs. Rarely/Never	1.08 (0.96–1.22)	1.01 (0.90–1.13)	1.02 (0.90–1.14)	1.02 (0.90–1.14)
	**Percentage of Time Spent Engaging in Intense PA** (>Half vs. Very Little/None) **			
≥Once/Week vs. Rarely/Never	**1.21 (1.09–1.35)**	*1.10 (1.00–1.22)*	*1.10 (0.99–1.21)*	*1.09 (0.99–1.20)*
1–3 Times/Month vs. Rarely/Never	*1.12 (0.98–1.27)*	1.03 (0.91–1.17)	1.05 (0.93–1.18)	1.06 (0.94–1.20)

Model 1: unadjusted; Model 2: adjusted for sociodemographic factors (age, educational attainment, household income, employment status, marital status); Model 3: Model 2 + health behaviors and characteristics (smoking status, alcohol usage, body mass index); Model 4: Model 3 + enjoyment of PA. Italicized values indicate statistical significance at a two-sided *p*-value < 0.10. Bolded values indicate statistical significance at a two-sided *p*-value < 0.05. Abbreviations: CI (confidence interval); PA (physical activity). Missing values: educational attainment = 1; household income = 3. * 118 of the 1558 participants were lost to follow-up and did not provide adulthood PA data. ** Percentage of time spent engaging in intense PA was assessed among the 817 participants who engaged in any minutes of PA per week, on average.

**Table 5:** Prevalence ratios for engagement in physical activity (PA) for participants by latent class of hair maintenance behaviors/ hair product usage in the past 12 months: Participants with more hair product use compared to participants with less hair product use, Study of Environment, Lifestyle and Fibroids (*N* = 1440 *).

Latent Classes of Hair Maintenance Behaviors/Hair Product Use	Prevalence Ratio (95% CI)			
	Model 1	Model 2	Model 3	Model 4
	**Participation in Leisure-time PA (yes vs. no)**			
Class 3 vs. Class 1	**1.15 (1.03–1.28)**	*1.10 (0.99–1.23)*	*1.10 (0.98–1.22)*	1.09 (0.98–1.21)
Class 2 vs. Class 1	**1.15 (1.03–1.28)**	1.08 (0.97–1.20)	1.07 (0.96–1.19)	1.09 (0.98–1.20)
	≥150 Minutes/Week of Leisure-time PA (yes vs. no)			
Class 3 vs. Class 1	**1.36 (1.12–1.65)**	**1.26 (1.04–1.53)**	**1.24 (1.02–1.50)**	**1.23 (1.02–1.49)**
Class 2 vs. Class 1	**1.23 (1.01–1.50)**	1.12 (0.92–1.37)	1.11 (0.91–1.35)	1.15 (0.94–1.39)
	**Percentage of Time Spent Engaging in Intense PA** (≤Half vs. Very Little/None) **			
Class 3 vs. Class 1	1.07 (0.95–1.21)	0.99 (0.88–1.11)	0.98 (0.87–1.10)	0.98 (0.87–1.10)
Class 2 vs. Class 1	1.05 (0.93–1.18)	0.99 (0.89–1.11)	1.01 (0.90–1.13)	1.01 (0.91–1.13)
	**Percentage of Time Spent Engaging in Intense PA** (>Half vs. Very Little/None) **			
Class 3 vs. Class 1	**1.17 (1.02–1.33)**	1.06 (0.94–1.19)	1.05 (0.94–1.18)	1.06 (0.94–1.19)
Class 2 vs. Class 1	1.10 (0.96–1.26)	1.00 (0.89–1.13)	1.01 (0.90–1.13)	1.02 (0.91–1.15)

Model 1: unadjusted; Model 2: adjusted for sociodemographic factors (age, educational attainment, household income, employment status, marital status); Model 3: Model 2 + health behaviors and characteristics (smoking status, alcohol usage, body mass index); Model 4: Model 3 + enjoyment of PA. Italicized values indicate statistical significance at a two-sided *p*-value < 0.10. Bolded values indicate statistical significance at a two-sided *p*-value < 0.05. Class 1: High Styling Product Use/ Low Use Other Products; Class 2: High Styling Product Use/Medium Shampoo and Conditioner Use; and Class 3: High Styling Product, Growth/Moisturizing Product, and Shampoo Use. Abbreviations: CI (confidence interval); PA (physical activity). Missing values: educational attainment = 1; household income = 3; * 118 of the 1558 participants were lost to follow-up and did not provide adulthood PA data. ** Percentage of time spent engaging in intense PA was assessed among the 817 participants who engaged in any minutes of PA per week, on average.

## Supplementary Materials

The following are available online at https://www.mdpi.com/1660-4601/17/24/9254/s1, Table S1: Bivariate associations between chemical relaxer and leave-in conditioner use; Table S2: Adulthood characteristics of participants by adulthood chemical relaxer use, SELF, *N* = 1440 *; Table S3: Adulthood characteristics of participants by adulthood leave-in conditioner use, SELF, *N* = 1440 *; Table S4: Adulthood characteristics of participants by latent class of hair maintenance behaviors/hair product use in adulthood, SELF, *N* = 1440 *; Table S5: Prevalence ratios for engagement in physical activity (PA) for participants by chemical hair product use or latent class of hair maintenance behaviors in the past 12 months, stratified by enjoyment of PA: Participants with more hair product use/hair maintenance compared to participants with less hair product use/hair maintenance, Study of Environment, Lifestyle and Fibroids (*N* = 1440 *); Table S6: Prevalence ratios for engagement in physical activity (PA) for participants by chemical hair product use or latent class of hair maintenance behaviors in the past 12 months, stratified by chemical relaxer use: Participants with more hair product use/hair maintenance compared to participants with less hair product use/hair maintenance, Study of Environment, Lifestyle and Fibroids (*N* = 1440 *); Table S7: Prevalence ratios for engagement in physical activity (PA) for participants by chemical hair product use or latent class of hair maintenance behaviors in the past 12 months, stratified by age group: Participants with more hair product use/hair maintenance compared to participants with less hair product use/hair maintenance, Study of Environment, Lifestyle and Fibroids (*N* = 1440 *); Table S8: Prevalence ratios for engagement in physical activity (PA) for participants by chemical hair product use or latent class of hair maintenance behaviors in the past 12 months, stratified by annual household income: Participants with more hair product use/hair maintenance compared to participants with less hair product use/hair maintenance, Study of Environment, Lifestyle and Fibroids (*N* = 1440 *); Table S9: Prevalence ratios for engagement in physical activity (PA) for participants by chemical hair product use or latent class of hair maintenance behaviors in the past 12 months, stratified by obesity status: Participants with more hair product use/hair maintenance compared to participants with less hair product use/hair maintenance, Study of Environment, Lifestyle and Fibroids (*N* = 1440 *).
