# Supplementary material for: Hair Maintenance and Chemical Hair Product Usage as Barriers to Physical Activity in Childhood and Adulthood among African American Women
Source: Int J Environ Res Public Health. 2020 Dec 10;17(24):9254. doi: 10.3390/ijerph17249254 (PMC7762987; doi:10.3390/ijerph17249254)
Supplement: Supplementary file 1 [file ijerph-17-09254-s001.pdf]

Supplemental Table S1. Bivariate associations between chemical relaxer and leave-in conditioner use

**Leave-in Conditioner Use (%)**	**Chemical Relaxer Use (%)**			**Total (%)**
	≥ Twice/Year	Once/Year	Rarely/Never	
≥Once/Week	7.0	2.6	14.3	23.9
1-3 Times/Month	5.0	1.9	12.8	19.7
Rarely/Never	17.6	7.3	31.5	56.4
**Total (%)**	29.6	11.8	58.6	100

X^2 p-value = 0.13

Supplemental Table S2. Adulthood characteristics of participants by adulthood chemical relaxer use, SELF, N=1,440*

	Chemical Relaxer/Straightener Use Mean ± SD or %		
	**≥ Twice/Year n=426**	**Once/Year n=171**	**Rarely/Never n=843**
**Sociodemographic Characteristics**			
Age, years (mean ± SD)	33 ± 3.3	32 ± 3.6	33 ± 3.5
Educational Attainment			
≤High School or GED	16	31	12
Some College/Associate or Technical Degree	58	50	48
≥ Bachelor's Degree	26	19	40
Total Annual Household Income			
<$20,000	40	58	29
$20,000-$50,000	37	29	42
>$50,000	23	13	29
Current Full- or Part-Time Employment (yes)	77	66	81
Marital Status			
Married/Living with Partner	43	34	41
Divorced/Separated/Widowed	19	23	19
Never Married	39	43	40
**Health Behaviors and Characteristics**			
BMI (kg/mg^2)			
Underweight/Normal (<25.0)	12	18	17
Overweight (25.0 to 29.9)	20	15	20
Obese Class I (30.0-34.9)	18	17	21
Obese Classes II and III (≥35.0)	50	50	42
Smoking Status			
Never	63	60	75
Former	12	8	8
Current	25	32	17
Alcohol Use (Past 12 Months)			
None	12	15	15
Moderate	64	58	70

Heavy	24	26	15
Good/Very Good/Excellent General Physical Health (Yes)	82	83	86
Enjoyment of Physical Activity			
Not at All/ A Little/ Somewhat	60	51	56
Quite a Bit/Very Much	40	49	44
**Physical Activity (PA) in the Past 12 Months**			
Participation in regular leisure time PA (sports, took exercise class/did other activities (e.g., biking, hiking, dancing)) (yes)	53	57	62
≥150 Minutes/Week of Regular Leisure-time Physical Activity (not including walking) (yes)	26	32	32
Percentage of Time Spent Engaging in Intense Physical Activity **			
> Half	33	33	48
≤ Half	52	54	40
Very Little/None	15	14	12

Abbreviations: SELF (Study of Environment, Lifestyle and Fibroids); SD (Standard Deviation); GED (General Education Development); BMI (Body Mass Index); PA (Physical Activity)

Note: Percentages may not sum to 100 due to missing values and rounding.

Missing Values: educational attainment= 1; household income= 2; general health= 9

*118 of the 1,558 participants were lost to follow-up and did not provide adulthood PA data.

** Percentage of time spent engaging in intense PA was assessed among the 817 participants who engaged in any minutes of PA per week, on average.

Supplemental Table S3. Adulthood characteristics of participants by adulthood leave-in conditioner use, SELF, N=1,440*

	Leave-in Conditioner Use Mean ± SD or %		
	**≥ Once/Week n=345**	**1-3 Times/Month n=284**	**Rarely/Never n=811**
**Sociodemographic Characteristics**			
Age, years (mean ± SD)	33 ± 3.3	33 ± 3.4	32 ± 3.5
Educational Attainment			
≤High School or GED	10	9	20
Some College/Associate or Technical Degree	50	50	52
≥ Bachelor's Degree	40	41	28
Total Annual Household Income			
<$20,000	31	27	40
$20,000-$50,000	41	43	37
>$50,000	28	30	23
Current Full- or Part-Time Employment (yes)	77	85	76
Marital Status			
Married/Living with Partner	38	43	41
Divorced/Separated/Widowed	23	19	18
Never Married	40	38	41
**Health Behaviors and Characteristics**			
BMI (kg/mg^2)			
Underweight/Normal (<25.0)	13	18	16
Overweight (25.0 to 29.9)	21	21	18
Obese Class I (30.0-34.9)	23	22	18
Obese Classes II and III (≥35.0)	43	39	48
Smoking Status			
Never	73	76	66
Former	7	10	10
Current	20	13	24
Alcohol Use (Past 12 Months)			
None	12	12	16
Moderate	68	75	63

Heavy	20	12	21
Good/Very Good/Excellent General Physical Health (Yes)	86	85	84
Enjoyment of Physical Activity			
Not at All/ A Little/ Somewhat	55	57	57
Quite a Bit/Very Much	45	43	43
**Physical Activity (PA) in the Past 12 Months**			
Participation in regular leisure time PA (sports, took exercise class/did other activities (e.g., biking, hiking, dancing)) (yes)	63	64	55
≥150 Minutes/Week of Regular Leisure-time Physical Activity (not including walking) (yes)	34	35	27
Percentage of Time Spent Engaging in Intense Physical Activity **			
> Half	49	43	39
≤ Half	43	45	45
Very Little/None	8	11	16

Abbreviations: SELF (Study of Environment, Lifestyle, and Fibroids); SD (Standard Deviation); GED (General Education Development); BMI (Body Mass Index); PA (Physical Activity)

Note: Percentages may not sum to 100 due to missing values and rounding.

Missing Values: educational attainment= 1; household income= 2; general health= 9

*118 of the 1,558 participants were lost to follow-up and did not provide adulthood PA data.

** Percentage of time spent engaging in intense PA was assessed among the 817 participants who engaged in any minutes of PA per week, on average.

Supplemental Table S4. Adulthood characteristics of participants by latent class of hair maintenance behaviors/hair product use in adulthood, SELF, N=1,440*

	Latent Class of Hair Maintenance Behaviors/ Hair Product Use Mean ± SD or %		
	**Class 1 n=512**	**Class 2 n=485**	**Class 3 n=443**
**Sociodemographic Characteristics**			
Age, years (mean ± SD)	33 ± 3.5	32 ± 3.4	32 ± 3.3
Educational Attainment			
≤High School or GED	22	10	13
Some College/Associate or Technical Degree	52	51	51
≥ Bachelor's Degree	26	38	36
Total Annual Household Income			
<$20,000	43	28	36
$20,000-$50,000	36	44	37
>$50,000	21	28	28
Current Full- or Part-Time Employment (yes)	74	83	76
Marital Status			
Married/Living with Partner	40	42	40
Divorced/Separated/Widowed	18	19	21
Never Married	42	39	39
**Health Behaviors and Characteristics**			
BMI (kg/mg^2)			
Underweight/Normal (<25.0)	16	17	13
Overweight (25.0 to 29.9)	19	19	20
Obese Class I (30.0-34.9)	20	19	20
Obese Classes II and III (≥35.0)	45	44	47
Smoking Status			
Never	64	75	70
Former	8	10	9
Current	27	15	21
Alcohol Use (Past 12 Months)			
None	17	12	14

Moderate	61	74	66
Heavy	22	14	21
Good/Very Good/Excellent General Physical Health (Yes)	83	87	84
Enjoyment of Physical Activity			
Not at All/ A Little/ Somewhat	56	58	54
Quite a Bit/Very Much	44	42	46
**Physical Activity (PA) in the Past 12 Months**			
Participation in regular leisure time PA (sports, took exercise class/did other activities (e.g., biking, hiking, dancing)) (yes)	54	62	62
≥150 Minutes/Week of Regular Leisure-time Physical Activity (not including walking) (yes)	26	32	35
Percentage of Time Spent Engaging in Intense Physical Activity **			
> Half	37	43	48
≤ Half	47	45	42
Very Little/None	16	13	11

Abbreviations: SELF (Study of Environment, Lifestyle, and Fibroids); SD (Standard Deviation); GED (General Education Development); BMI (Body Mass Index); PA (Physical Activity)

Note: Percentages may not sum to 100 due to missing values and rounding.

Class 1: High Styling Product Use/ Low Use Other Products

Class 2: High Styling Product Use/ Medium Shampoo & Conditioners

Class 3: High Styling Product, Growth/Moisturizing Product, and Shampoo Use

Missing Values: educational attainment= 1; household income= 2; general health= 9

*118 of the 1,558 participants were lost to follow-up and did not provide adulthood PA data.

** Percentage of time spent engaging in intense PA was assessed among the 817 participants who engaged in any minutes of PA per week, on average.

Supplemental Table S5. Prevalence ratios for engagement in physical activity (PA) for participants by chemical hair product use or latent class of hair maintenance behaviors in the past 12 months, stratified by enjoyment of PA: Participants with more hair product use/hair maintenance compared to participants with less hair product use/hair maintenance, Study of Environment, Lifestyle and Fibroids (N=1,440*)

Chemical Hair Product OR Latent Classes of Hair Maintenance Behaviors/ Hair Product Use	Enjoyment of PA (Quite a Bit/Very Much)							
	**No** (n=812)	**Yes** (n=628)	**No** (n=812)	**Yes** (n=628)	**No** (n=229)	**Yes** (n=241)	**No** (n=195)	**Yes** (n=258)
	**Prevalence Ratio (95% CI)**							
	**Participation in Leisure-time PA (Yes vs. No)**		**≥150 Minutes/Week of Leisure-time PA (Yes vs. No)**		**Percentage of Time Spent Engaging in Intense PA ** (≤ Half vs. Very Little/None)**		**Percentage of Time Spent Engaging in Intense PA ** (> Half vs. Very Little/None)**	
**Chemical Relaxer**								
≥ Twice/Year vs. Rarely/Never	0.85 (0.72-1.01)	0.95 (0.85-1.06)	0.75 (0.54-1.04)	1.01 (0.82-1.25)	0.95 (0.80-1.13)	1.04 (0.91-1.19)	0.88 (0.70-1.11)	0.92 (0.80-1.06)
Once/Year vs. Rarely/Never	0.92 (0.71-1.18)	1.05 (0.91-1.22)	0.97 (0.62-1.54)	1.14 (0.87-1.48)	1.14 (0.92-1.42)	1.11 (0.92-1.35)	1.13 (0.82-1.57)	0.98 (0.80-1.20)
**Leave-in Conditioner**								
≥Once/Week vs. Rarely/Never	0.99 (0.83-1.19)	**1.14 (1.03-1.26)**	1.00 (0.72-1.40)	1.17 (0.95-1.43)	1.03 (0.87-1.22)	1.06 (0.93-1.22)	0.98 (0.81-1.18)	1.10 (0.98-1.23)
1-3 Times/Month vs. Rarely/Never	1.10 (0.93-1.31)	1.06 (0.94-1.19)	1.18 (0.86-1.63)	1.16 (0.93-1.46)	0.97 (0.81-1.16)	1.05 (0.90-1.23)	1.05 (0.86-1.27)	1.06 (0.91-1.24)
**Latent Classes**								
Class 3 vs. Class 1	1.10 (0.91-1.33)	1.06 (0.95-1.18)	1.24 (0.88-1.76)	1.18 (0.95-1.48)	0.91 (0.76-1.10)	1.04 (0.89-1.21)	1.04 (0.85-1.28)	1.07 (0.93-1.23)
Class 2 vs. Class 1	1.13 (0.95-1.36)	1.01 (0.90-1.14)	1.07 (0.76-1.52)	1.17 (0.93-1.47)	0.91 (0.77-1.08)	1.11 (0.96-1.28)	0.94 (0.76-1.16)	1.12 (0.97-1.29)

Models are adjusted for sociodemographic factors (age, educational attainment, household income, employment status, marital status), and health behaviors/characteristics (smoking status, alcohol usage, body mass index category).
Bolded values indicate statistical significance at a two-sided p-value < 0.05.
Class 1: High Styling Product Use/ Low Use Other Products
Class 2: High Styling Product Use/ Medium Shampoo & Conditioners
Class 3: High Styling Product, Growth/Moisturizing Product, and Shampoo Use
Abbreviations: CI (Confidence Interval); PA (physical activity)
Missing values: educational attainment= 1; household income= 3
* 118 of the 1,558 participants were lost to follow-up and did not provide adulthood PA data.
** Percentage of time spent engaging in intense PA was assessed among the 817 participants who engaged in any minutes of PA per week, on average.
Note: No interaction terms had a two-sided p-value <0.10.

Supplemental Table S6. Prevalence ratios for engagement in physical activity (PA) for participants by chemical hair product use or latent class of hair maintenance behaviors in the past 12 months, stratified by chemical relaxer use: Participants with more hair product use/hair maintenance compared to participants with less hair product use/hair maintenance, Study of Environment, Lifestyle and Fibroids (N=1,440*)

	**Chemical Relaxer Use**							
	**No** (n=843)	**Yes** (n=597)	**No** (n=843)	**Yes** (n=597)	**No** (n=263)	**Yes** (n=207)	**No** (n=306)	**Yes** (n=147)
**Chemical Hair Product OR Latent Classes of Hair Maintenance Behaviors/ Hair Product Use**	**Prevalence Ratio (95% CI)**							
	**Participation in Leisure-time PA (Yes vs. No)**		**≥150 Minutes/Week of Leisure-time PA (Yes vs. No)**		**Percentage of Time Spent Engaging in Intense PA ** (≤ Half vs. Very Little/None)**		**Percentage of Time Spent Engaging in Intense PA ** (> Half vs. Very Little/None)**	
**Leave-in Conditioner**								
≥Once/Week vs. Rarely/Never	1.05 (0.93-1.18)	1.12 (0.95-1.31)	1.14 (0.91-1.43)	1.18 (0.89-1.56)	1.10 (0.96-1.26)	0.95 (0.80-1.12)	**1.17 (1.06-1.30)**	0.89 (0.69-1.15)
1-3 Times/Month vs. Rarely/Never	1.10 (0.97-1.25)	1.04 (0.85-1.26)	1.24 (0.99-1.55)	1.07 (0.77-1.49)	0.97 (0.82-1.14)	1.11 (0.94-1.30)	1.06 (0.93-1.22)	1.18 (0.90-1.54)
**Latent Classes**								
Class 3 vs. Class 1	1.12 (0.99-1.27)	1.04 (0.88-1.24)	**1.43 (1.11-1.85)**	1.03 (0.76-1.38)	0.93 (0.79-1.09)	1.03 (0.86-1.24)	1.08 (0.95-1.23)	1.02 (0.78-1.34)
Class 2 vs. Class 1	1.11 (0.98-1.27)	1.04 (0.88-1.23)	**1.34 (1.04-1.73)**	0.94 (0.69-1.27)	0.91 (0.79-1.06)	1.15 (0.97-1.36)	1.01 (0.88-1.16)	1.10 (0.87-1.39)

Models are adjusted for sociodemographic factors (age, educational attainment, household income, employment status, marital status), health behaviors and characteristics (smoking status, alcohol usage, body mass index), and enjoyment of PA.
Bolded values indicate statistical significance at a two-sided p-value < 0.05.
Class 1: High Styling Product Use/ Low Use Other Products
Class 2: High Styling Product Use/ Medium Shampoo & Conditioners
Class 3: High Styling Product, Growth/Moisturizing Product, and Shampoo Use
Abbreviations: CI (Confidence Interval); PA (physical activity)
Missing values: educational attainment= 1; household income= 3
* 118 of the 1,558 participants were lost to follow-up and did not provide adulthood PA data.
** Percentage of time spent engaging in intense PA was assessed among the 817 participants who engaged in any minutes of PA per week, on average.
Note: No interaction terms had a two-sided p-value <0.10.

Supplemental Table S7. Prevalence ratios for engagement in physical activity (PA) for participants by chemical hair product use or latent class of hair maintenance behaviors in the past 12 months, stratified by age group: Participants with more hair product use/hair maintenance compared to participants with less hair product use/hair maintenance, Study of Environment, Lifestyle and Fibroids (N=1,440*)

	**Age Group**							
	**<33 Years** (n=702)	**≥33 Years** (n=738)	**<33 Years** (n=702)	**≥33 Years** (n=738)	**<33 Years** (n=239)	**≥33 Years** (n=231)	**<33 Years** (n=217)	**≥33 Years** (n=236)
**Chemical Hair Product OR Latent Classes of Hair Maintenance Behaviors/ Hair Product Use**	**Prevalence Ratio (95% CI)**							
	**Participation in Leisure-time PA (Yes vs. No)** [LIC]		**≥150 Minutes/Week of Leisure-time PA (Yes vs. No)** [CR]		**Percentage of Time Spent Engaging in Intense PA ** (≤ Half vs. Very Little/None)**		**Percentage of Time Spent Engaging in Intense PA ** (> Half vs. Very Little/None)**	
**Chemical Relaxer**								
≥ Twice/Year vs. Rarely/Never	0.95 (0.82-1.09)	0.86 (0.75-0.99)	1.13 (0.89-1.43)	0.71 (0.53-0.93)	1.01 (0.88-1.16)	1.00 (0.85-1.18)	0.86 (0.71-1.05)	0.95 (0.81-1.12)
Once/Year vs. Rarely/Never	0.98 (0.81-1.19)	1.04 (0.87-1.26)	1.07 (0.77-1.49)	1.20 (0.86-1.67)	1.03 (0.83-1.28)	1.17 (0.97-1.41)	1.06 (0.84-1.35)	0.96 (0.74-1.23)
**Leave-in Conditioner**								
≥Once/Week vs. Rarely/Never	1.09 (0.95-1.26)	1.06 (0.94-1.21)	0.92 (0.71-1.19)	1.36 (1.06-1.73)	1.04 (0.91-1.20)	1.06 (0.91-1.24)	1.03 (0.89-1.19)	1.14 (1.00-1.30)
1-3 Times/Month vs. Rarely/Never	**1.21 (1.05-1.39)**	0.96 (0.82-1.13)	1.09 (0.85-1.39)	1.24 (0.92-1.66)	1.01 (0.87-1.17)	1.02 (0.84-1.24)	1.05 (0.89-1.25)	1.07 (0.89-1.27)
**Latent Classes**								
Class 3 vs. Class 1	1.15 (0.99-1.34)	1.04 (0.91-1.19)	1.20 (0.92-1.57)	1.25 (0.96-1.63)	1.04 (0.89-1.22)	0.92 (0.76-1.11)	1.10 (0.92-1.31)	1.05 (0.90-1.23)
Class 2 vs. Class 1	**1.22 (1.05-1.42)**	0.98 (0.85-1.13)	1.15 (0.88-1.50)	1.08 (0.82-1.44)	0.99 (0.84-1.18)	1.02 (0.87-1.18)	1.03 (0.86-1.22)	1.00 (0.85-1.18)

Models are adjusted for sociodemographic factors (age [years], educational attainment, household income, employment status, marital status), health behaviors and characteristics (smoking status, alcohol usage, body mass index category), and enjoyment of PA.
Bolded values indicate statistical significance at a two-sided p-value < 0.05.
Class 1: High Styling Product Use/ Low Use Other Products
Class 2: High Styling Product Use/ Medium Shampoo & Conditioners
Class 3: High Styling Product, Growth/Moisturizing Product, and Shampoo Use
Abbreviations: CI (Confidence Interval); PA (physical activity)
Missing values: educational attainment= 1; household income= 3
* 118 of the 1,558 participants were lost to follow-up and did not provide adulthood PA data.
** Percentage of time spent engaging in intense PA was assessed among the 817 participants who engaged in any minutes of PA per week, on average.
[LIC] Interaction term for leave-in conditioner*age group two-sided p-value <0.10.
[CR] Interaction term for chemical relaxer*age group two-sided p-value <0.05.

Supplemental Table S8. Prevalence ratios for engagement in physical activity (PA) for participants by chemical hair product use or latent class of hair maintenance behaviors in the past 12 months, stratified by annual household income: Participants with more hair product use/hair maintenance compared to participants with less hair product use/hair maintenance, Study of Environment, Lifestyle and Fibroids (N=1,440*)

Chemical Hair Product OR Latent Classes of Hair Maintenance Behaviors/ Hair Product Use	Annual Household Income							
	**≤$50,000** (n=1,074)	**>$50,000** (n=364)	**≤$50,000** (n=1,074)	**>$50,000** (n=364)	**≤$50,000** (n=357)	**>$50,000** (n=113)	**≤$50,000** (n=300)	**>$50,000** (n=153)
	**Prevalence Ratio (95% CI)**							
	**Participation in Leisure-time PA (Yes vs. No)**		**≥150 Minutes/Week of Leisure-time PA (Yes vs. No)**		**Percentage of Time Spent Engaging in Intense PA ** (≤ Half vs. Very Little/None)**		**Percentage of Time Spent Engaging in Intense PA ** (> Half vs. Very Little/None)**	
**Chemical Relaxer**								
≥ Twice/Year vs. Rarely/Never	0.89 (0.79-1.01)	0.92 (0.78-1.08)	0.86 (0.69-1.07)	0.94 (0.69-1.29)	0.97 (0.85-1.11)	1.06 (0.91-1.24)	**0.81 (0.67-0.98)**	1.02 (0.88-1.18)
Once/Year vs. Rarely/Never	0.97 (0.84-1.14)	1.06 (0.83-1.36)	0.96 (0.74-1.25)	1.48 (0.92-2.38)	1.06 (0.90-1.26)	**1.22 (1.03-1.44)**	0.93 (0.74-1.18)	1.10 (0.98-1.25)
**Leave-in Conditioner**								
≥Once/Week vs. Rarely/Never	1.02 (0.90-1.15)	**1.23 (1.07-1.41)**	1.12 (0.90-1.39)	1.20 (0.88-1.63)	1.04 (0.90-1.20)	0.98 (0.86-1.12)	1.16 (1.00-1.35)	1.02 (0.91-1.14)
1-3 Times/Month vs. Rarely/Never	1.09 (0.96-1.25)	1.11 (0.94-1.31)	1.20 (0.95-1.52)	1.22 (0.90-1.66)	1.06 (0.92-1.22)	0.89 (0.73-1.10)	1.15 (0.96-1.38)	0.94 (0.81-1.08)
**Latent Classes**								
Class 3 vs. Class 1	1.09 (0.95-1.24)	1.08 (0.93-1.27)	1.35 (1.07-1.69)	1.00 (0.72-1.40)	1.02 (0.88-1.19)	0.90 (0.77-1.06)	1.17 (0.99-1.40)	0.92 (0.82-1.04)
Class 2 vs. Class 1	1.14 (1.00-1.29)	0.98 (0.83-1.16)	1.25 (0.99-1.59)	0.98 (0.71-1.35)	1.05 (0.91-1.20)	0.93 (0.80-1.08)	1.06 (0.89-1.28)	0.94 (0.84-1.06)

Models are adjusted for sociodemographic factors (age, educational attainment, employment status, marital status), health behaviors and characteristics (smoking status, alcohol usage, body mass index category, and enjoyment of PA.
Bolded values indicate statistical significance at a two-sided p-value < 0.05.
Class 1: High Styling Product Use/ Low Use Other Products
Class 2: High Styling Product Use/ Medium Shampoo & Conditioners
Class 3: High Styling Product, Growth/Moisturizing Product, and Shampoo Use
Abbreviations: CI (Confidence Interval); PA (physical activity)
Missing values: educational attainment= 1; household income= 3
* 118 of the 1,558 participants were lost to follow-up and did not provide adulthood PA data.
** Percentage of time spent engaging in intense PA was assessed among the 817 participants who engaged in any minutes of PA per week, on average.
Note: No interaction terms had a two-sided p-value <0.10.

Supplemental Table S9. Prevalence ratios for engagement in physical activity (PA) for participants by chemical hair product use or latent class of hair maintenance behaviors in the past 12 months, stratified by obesity status: Participants with more hair product use/hair maintenance compared to participants with less hair product use/hair maintenance, Study of Environment, Lifestyle and Fibroids (N=1,440*)

	**Obesity Status**							
	**Non-Obese** (n=504)	**Obese** (n=936)	**Non-Obese** (n=504)	**Obese** (n=936)	**Non-Obese** (n=178)	**Obese** (n=292)	**Non-Obese** (n=155)	**Obese** (n=298)
**Chemical Hair Product OR Latent Classes of Hair Maintenance Behaviors/ Hair Product Use**	**Prevalence Ratio (95% CI)**							
	**Participation in Leisure-time PA (Yes vs. No)**		**≥150 Minutes/Week of Leisure-time PA (Yes vs. No)**		**Percentage of Time Spent Engaging in Intense PA ** (≤ Half vs. Very Little/None)**		**Percentage of Time Spent Engaging in Intense PA ** (> Half vs. Very Little/None) [LC]**	
**Chemical Relaxer**								
≥ Twice/Year vs. Rarely/Never	0.97 (0.82-1.15)	**0.87 (0.77-0.98)**	0.99 (0.71-1.38)	0.88 (0.71-1.09)	1.14 (0.96-1.35)	0.94 (0.83-1.08)	1.03 (0.82-1.29)	**0.83 (0.71-0.97)**
Once/Year vs. Rarely/Never	0.88 (0.69-1.12)	1.05 (0.90-1.24)	0.84 (0.53-1.32)	1.18 (0.90-1.56)	1.12 (0.86-1.47)	1.06 (0.90-1.25)	1.05 (0.75-1.46)	1.01 (0.83-1.23)
**Leave-in Conditioner**								
≥Once/Week vs. Rarely/Never	**1.20 (1.03-1.41)**	1.02 (0.90-1.15)	1.30 (0.96-1.76)	1.07 (0.86-1.33)	1.07 (0.91-1.25)	1.00 (0.87-1.14)	1.20 (1.00-1.43)	1.03 (0.92-1.15)
1-3 Times/Month vs. Rarely/Never	**1.19 (1.01-1.42)**	1.03 (0.91-1.18)	**1.48 (1.07-2.04)**	1.06 (0.84-1.34)	1.04 (0.84-1.30)	0.98 (0.85-1.12)	1.04 (0.83-1.30)	1.04 (0.90-1.20)
**Latent Classes**								
Class 3 vs. Class 1	1.17 (0.99-1.38)	1.04 (0.91-1.18)	**1.48 (1.07-2.05)**	1.12 (0.89-1.41)	1.10 (0.91-1.32)	0.92 (0.79-1.06)	1.22 (0.96-1.55)	0.97 (0.85-1.10)
Class 2 vs. Class 1	1.08 (0.91-1.28)	1.09 (0.96-1.24)	1.19 (0.84-1.68)	1.12 (0.89-1.41)	1.04 (0.85-1.28)	0.98 (0.86-1.12)	1.02 (0.78-1.33)	0.99 (0.88-1.12)

Models are adjusted for sociodemographic factors (age, educational attainment, household income, employment status, marital status), health behaviors and characteristics (smoking status, alcohol usage) and enjoyment of PA.
Bolded values indicate statistical significance at a two-sided p-value < 0.05.
Class 1: High Styling Product Use/ Low Use Other Products
Class 2: High Styling Product Use/ Medium Shampoo & Conditioners
Class 3: High Styling Product, Growth/Moisturizing Product, and Shampoo Use
Abbreviations: CI (Confidence Interval); PA (physical activity)
Missing values: educational attainment= 1; household income= 3
* 118 of the 1,558 participants were lost to follow-up and did not provide adulthood PA data.
** Percentage of time spent engaging in intense PA was assessed among the 817 participants who engaged in any minutes of PA per week, on average.
[LC] Interaction term for latent class*age group two-sided p-value <0.10.
